# Obviation of dyslipidemia by garlic oil and its organosulfur compound, diallyl disulphide, in experimental animals

**DOI:** 10.1016/j.sjbs.2021.12.025

**Published:** 2021-12-20

**Authors:** Syed Mohammed Basheeruddin Asdaq, Farhana Yasmin, Abdulkhaliq J. Alsalman, Mohammed Al mohaini, Mehnaz Kamal, Maitham A. Al Hawaj, Khaled J. Alsalman, Mohd. Imran, Nagaraja Sreeharsha

**Affiliations:** a Department of Pharmacy Practice, College of Pharmacy, AlMaarefa University, Diriyah, 13713 Riyadh, Saudi Arabia; b Department of Mathematics, College of Applied Sciences, AlMaarefa University, Dariyah, 13713 Riyadh, Saudi Arabia; c Department of Clinical Pharmacy, Faculty of Pharmacy, Northern Border University, Rafha 91911, Saudi Arabia; d Basic Sciences Department, College of Applied Medical Sciences, King Saud bin Abdulaziz University for Health Sciences, Alahsa, 31982, Saudi Arabia; e King Abdullah International Medical Research Center, Alahsa, 31982, Saudi Arabia; f Department of Pharmaceutical Chemistry, College of Pharmacy, Prince Sattam Bin Abdulaziz University, Al-Kharj 11942, Saudi Arabia; g Department of Pharmacy Practice, College of Clinical Pharmacy, King Faisal University, Ahsa 31982, Saudi Arabia; h Pharmaceutical Care Department, Albatha General Hospital, Alodaid 36636, Saudi Arabia; i Department of Pharmaceutical Chemistry, Faculty of Pharmacy, Northern Border University, Rafha 91911, Saudi Arabia; j Department of Pharmaceutical Sciences, College of Clinical Pharmacy, King Faisal University, Al-Ahsa 31982, Saudi Arabia; k Department of Pharmaceutics, Vidya Siri College of Pharmacy, Off Sarjapura Road, Bangalore 560035, India

**Keywords:** Garlic oil, Diallyl disulphide, High fat diet, Antihyperlipidemic, Antioxidant

## Abstract

**Background and objectives:**

Garlic and its number of preparations are known to be effective for treatment of dyslipidemia, but the data about the specific active constituents of the garlic on the possible therapeutic value is scarce. Therefore, the aim of this research was to evaluate the role of garlic oil (GO) and its active element, diallyl disulphide (DADS) for obviating dyslipidemia in animal model.

**Methods:**

High fat diet (HFD) was given to animals to induce dyslipidemia. Animals of HFD groups were fed with atherogenic diet for 15 days prior to treatment. Animals in their respective groups received vehicle, GO (50 and 100 mg/kg), and DADS (4.47 and 8.94 mg/kg) for five consecutive days. Lipid profiles were estimated in serum, oxidant/antioxidant and liver profile were measured in liver tissue homogenate (LTH).

**Results:**

Animals fed on HFD developed significant increase in the serum levels of triglycerides (TG), total cholesterol (TC), lactate dehydrogenase (LDL), malondialdehyde (MDA), glutathione peroxidase (GSHPx), glutathione (GSH), and glutathione disulfide (GSSG) that reduced significantly in groups that received GO and DADS treatments. Additionally, significant elevation in serum high density lipoprotein (HDL) level was observed in animals that received GO and DADS. Moreover, hepatic markers such as alkaline phosphatase (ALP), aspartate aminotransferase (AST), and alanine transferase (ALT), that were abnormally altered by high fat diet, were significantly restored to almost normal values with GO and DADS treatments. Also, antioxidants such as superoxide dismutase (SOD), catalase (CAT), ferric reducing antioxidant power (FRAP), and total thiol (SH) levels in LTH were increased significantly in GO and DADS treated groups. When compared to DADS, GO showed better therapeutic effectiveness in terms of antihyperlipidemic and antioxidant properties.

**Conclusion:**

In hyperlipidemic rats, garlic and its principal active component, diallyl disulphide, were effective in avoiding dyslipidemia and neutralizing reactive free radicals induced by a high fat diet. It's an intriguing observation that GO has a larger therapeutic influence than its active constituent, DADS. These findings suggest that other constituents, in addition to GO's DADS, are involved in the compound's synergistic antihyperlipidemic and antioxidant activities.

## 1 Introduction

In many regions of the world, cardiovascular morbidity is a leading cause of death. Anti-hyperlipidemic medications are already available to treat hyperlipidemia, but their use is limited due to the increased risk of side effects (Nasri and Shirzad, 2013). In comparison to synthetic drugs, plant-based therapies are thought to possess less undesired effects. There are number of studies that show an inverse relationship between a food that has vegetables and the prevalence of ischemic heart illness (Mozaffarian et al., 2003, Ruscica et al., 2021 Jun 27). As a result, it was thought useful to investigate the impact of a regularly used plant derived material and one of its main elements in obviating dyslipidemia using established animal models. Garlic (Allium sativum L.) and its different forms are known since long time as an excellent preventative and therapeutic medicinal agent in the traditional system (Asdaq and Inamdar, 2009, Adaki et al., 2014, Ribeiro et al., 2021 Mar 10). It is generally recognized in the form of spice and a home remedy for variety of disorders (Tripathi, 2009). Many studies have found that chronic garlic usage lowers plasma lipids (Asdaq et al., 2009), decreases pro-inflammatory cytokine production, and reduces platelet activation state (Sonia et al., 2004). Raw garlic, on the other hand, has a variety of harmful effects, including anemia, weight loss, and growth retardation (Ray et al., 2011). Garlic's outstanding biological properties include oxidative radical scavenging capability, cardioprotective qualities, and effectiveness as an adjunct in the treatment of a variety of malignancies, as evidenced by several research studies conducted in recent decades (Hayat et al., 2016). In addition, number of studies (Asdaq et al., 2021, Batiha et al., 2020) examined the therapeutic benefits of main elements contained in garlic preparations such as garlic oil, and powder.

Garlic has number of beneficial ingredients that include organosulfur substances in addition to saponins, and phenolic compounds (Wang et al., 2018). Organosulfur compound, diallyl disulfide (DADS), is one among the main active ingredients reported in the literature (Mansingh et al., 2018). In addition to DADS, diallyl thiosulfonate (allicin), diallyl sulfide (DAS), diallyl trisulfide (DATS), SAC, and S-allyl-cystein sulfoxide are all reported to be present in garlic oil with therapeutic efficacy (Mansingh et al., 2018). However, we selected DADS based on our experience of its therapeutic potential (Asdaq et al., 2021a, Asdaq et al., 2021b). Further, garlic oil (GO) is shown to be effective in reducing body weight by reducing low-density lipoprotein (LDL) (Yang et al., 2018). Therefore, it was our interest to explore the potential benefit of GO and its active constituent, DADS, in obviating dyslipidemia using animal models.

## 2 Materials and methods

### 2.1 Experimental animals

Sprague-Dawley rats (220–250 g) were utilized, and they were housed in an animal house that was kept in compliance with the standards and ethical requirements of the local ethics commission (Research committee, College of Pharmacy, AlMaarefa University, Riyadh, Saudi Arabia) that accepted our research project.

### 2.2 Experimental protocol

There were five groups each under NFD and HFD categories (n = 8). The rats of the NFD group received standard pellet supplied by standard supplier that had protein, oil, fibre, ash, and silica in a percentage of 22.10, 4.13, 3.15, 5.15 and 1.12, respectively. The HFD animals were given 68% normal fat diet in addition to dalda (30%) and cholesterol (2%) (Guido and Joseph, 1992; Asdaq et al., 2009) for 15 days prior to the commencement of treatment and during the period of administration of drugs (five days).

### 2.3 Experimental steps

Animals in group I to V received normal fat diet and treated orally with vehicle, GO (50 and 100 mg/kg) and DADS (4.47 and 8.94 mg/kg) (Asdaq et al., 2021), respectively for five days. Group VI, VII, VIII, IX and X of HFD category were similarly administered with vehicle, GO and DADS, respectively, for five days. The weights of the animals were taken at the beginning and before they were sacrificed, and the % change in weight was determined. Each animal's daily feed consumption was also recorded in grams per day (Asdaq, 2015).

### 2.4 Biochemical estimations in serum

A method described by Asdaq and Inamdar (2010) was used to estimate protein levels and GSHPx, GSH and GSSG. An autoanalyzer was employed to calculate TG, TC, HDL (El-Hazmi and Warsy, 2001). The LDL was calculated based on the amount of TC, HDL and TG with a formula described by Friedwald’s et al. (1972). In addition to this, AI was calculated based on the ratio between LDL and HDL (Bahramikia and Yazdanparast, 2008). Additionally, liver parameters such as AST, ALT, and ALP were measured.

### 2.5 Biochemical estimations in LTH

Livers were promptly separated from sacrificed rats, cleaned in normal saline, and soaked using filter paper. Liver tissue homogenate (LTH) was prepared in sucrose to estimate antioxidants. A method to measure TBARS, FRAP, SH groups assay, SOD and CAT described by Asdaq and Inamdar, 2010 was used in this study.

### 2.6 Data statistics

The values obtained in the study were statistically tested for determining the level of significance. Any comparison between the group where probability value was lower than 0.05 was termed as significant.

## 3 Results

### 3.1 Impact on lipid parameters

Table 1 explain the impact of five days of administration of garlic oil and DADS in their respective groups on lipid profile. HFD control animals showed significantly (*P* < 0.001) increased level of triglycerides, LDL, and total cholesterol in comparison with the normal control group. Similarly significant (*P* < 0.01) fall in the HDL cholesterol level was observed in high fat diet control animals compared to normal rats. Atherogenic index of animals under high fat diet was significantly (*P* < 0.001) high compared to animals kept on normal diet. Treatment of rats with GO, and DADS dose dependently caused significant (*P* < 0.05 with low dose and *P* < 0.01 at high dose) decrease in triglycerides (TG), total cholesterol (TC) and LDL cholesterol levels when compared to normal control group. Further, administration of GO and DADS, both low and high doses, significantly (*P* < 0.001) decreased the elevated TG, TC and LDL levels in the serum of animals who were fed with high fat diet signifying their antihyperlipidemic potential in hyperlipidemic animals. The results were further validated by atherogenic index that was significantly (*P* < 0.001) lowered in animals that received GO and DADs treatments.

**Table 1:** Lipid parameters.

Groups	TG	TC	HDL	LDH	AI
NC	68.28 ± 1.22	81.19 ± 1.21	34.89 ± 1.22	68.87 ± 1.66	1.97 ± 0.09
NGOL	61.22 ± 2.22*	69.22 ± 1.22^**^	39.98 ± 1.18*	57.54 ± 1.77*	1.43 ± 0.04^***^
NGOH	49.28 ± 1.33^**^	59.24 ± 1.23^**^	44.55 ± 1.66^***^	49.88 ± 1.55^***^	1.11 ± 0.03^***^
NDADSL	62.11 ± 1.45*	70.54 ± 1.32^**^	38.99 ± 1.44*	59.99 ± 1.43*	1.53 ± 0.09^***^
NDADSH	54.32 ± 1.11^**^	63.28 ± 1.11^**^	41.87 ± 1.47^***^	51.11 ± 1.32^**^	1.22 ± 0.31^***^
HFDC	108.22 ± 1.28^aaa^	132.35 ± 1.32^aaa^	29.55 ± 1.88^aa^	122.23 ± 1.82^aaa^	4.13 ± 0.31^aaa^
HFGOL	89.11 ± 1.11^**^	98.24 ± 1.80^**^	36.32 ± 1.58^***^	86.16 ± 1.47^***^	2.37 ± 0.20^***^
HFGOH	74.32 ± 1.99^***^	88.23 ± 1.65^***^	48.98 ± 1.65^***^	63.20 ± 1.45^***^	1.29 ± 0.12^***^
HFDADSL	91.26 ± 1.56^**^	112.40 ± 1.16^**^	37.65 ± 1.53^**^	91.99 ± 1.44^***^	2.44 ± 0.26^***^
HFDADSH	79.22 ± 1.26^***^	94.28 ± 1.098^**^	45.76 ± 1.46^***^	74.25 ± 1.32^***^	1.62 ± 0.22^***^

Lipid parameters measured in mg/kg; Values are given as mean ± SEM of eight rats; diallyl disulfide (DADS); NC: normal control; NGOL: normal Garlic oil low dose (50 mg/kg); NGOH: normal Garlic oil high dose (100 mg/kg); NDADSL: normal DADS low dose (4.47 mg/kg); NDADSH: normal DADS high dose (8.94 mg/kg); HFDC: high fat diet control; HFGOL: high fat Garlic oil low dose (50 mg/kg); HFGOH: high fat Garlic oil high dose (100 mg/kg); HFDADSL: high fat DADS low dose (4.47 mg/kg); HFDADSH: high fat DADS high dose (8.94 mg/kg); *P < 0.05; ^**^P < 0.01 and ^***^P < 0.001 normal diet fed treated groups Vs normal fat diet control and high fat diet treated Vs high fat diet control respectively. ^aaa^*P* < 0.001 NFD control Vs HFD control.

### 3.2 Impact on liver parameters, body weight change (%) and daily diet intake

In comparison to normal controls, rats treated with GO and DADS had significantly (*P* < 0.001) lower AST, ALT, and ALP levels. In addition, animals given high doses of GO and DADS had a significant (*P* < 0.01) reduction in daily diet intake (Table 2). A switch to a high-fat diet resulted in significant (*P* < 0.001) increases in liver enzymes, and body weight, as well as a decrease in daily diet intake. When compared to a high fat diet control, GO and DADS administration resulted in significant (*P* < 0.001) depletion of liver enzymes, and body weight, as well as an increase in daily diet intake (Table 2).

**Table 2:** Liver parameters.

Groups	ALP	AST	ALT	Percentage change in body weight	Diet intake (g/day)
NC	188.1 ± 9.8	25.1 ± 1.6	18.1 ± 1.3	10.6 ± 1.4	14.2 ± 1.2
NGOL	169.2 ± 6.5^**^	21.3 ± 1.8*	15.8 ± 1.4^**^	10.2 ± 1.3	14.3 ± 1.3
NGOH	161.5 ± 8.8^**^	14.5 ± 1.9^**^	13.4 ± 1.2^**^	11.4 ± 1.2	14.2 ± 1.1
NDADSL	176.7 ± 8.1*	22.1 ± 1.2*	15.6 ± 1.4^**^	10.9 ± 1.5	15.1 ± 1.4
NDADSH	165.1 ± 7.4^**^	17.2 ± 1.7^**^	13.8 ± 0.9^**^	11.6 ± 1.6	15.1 ± 1.3
HFDC	287.2 ± 10.3^aaa^	43.4 ± 2.2^aaa^	36.4 ± 1.6^aaa^	24.4 ± 1.8^aaa^	12.8 ± 1.6^aa^
HFGOL	251.1 ± 5.6*	36.5 ± 2.2^**^	31. ± 1.7*	19.2 ± 1.1*	11.5 ± 1.4
HFGOH	208.2 ± 11.2^***^	28.6 ± 2.3^**^	24.3 ± 1.0^**^	16.8 ± 1.6^**^	13.3 ± 1.3
HFDADSL	261.4 ± 9.5*	39.5 ± 1.8	32.3 ± 1.2*	21.3 ± 1.3*	12.4 ± 1.3
HFDADSH	217.7 ± 9.4^**^	30.2 ± 1.6*	26.5 ± 1.1*	15.5 ± 1.6^**^	13.3 ± 1.4

Values are given as mean ± SEM of eight rats; diallyl disulfide (DADS); NC: normal control; NGOL: normal Garlic oil low dose (50 mg/kg); NGOH: normal Garlic oil high dose (100 mg/kg); NDADSL: normal DADS low dose (4.47 mg/kg); NDADSH: normal DADS high dose (8.94 mg/kg); HFDC: high fat diet control; HFGOL: high fat Garlic oil low dose (50 mg/kg); HFGOH: high fat Garlic oil high dose (100 mg/kg); HFDADSL: high fat DADS low dose (4.47 mg/kg); HFDADSH: high fat DADS high dose (8.94 mg/kg); Aspartate aminotransferase: AST (IU/L); Alanine Aminotransferase: ALT (IU/L); Alkaline phosphatase: ALP (IU/L); *P < 0.05; ^**^P < 0.01 and ^***^P < 0.001 normal diet fed treated groups Vs normal fat diet control and high fat diet treated Vs high fat diet control respectively. ^aaa^*P* < 0.001 NFD control Vs HFD control.

### 3.3 Impact on oxidative parameters

As demonstrated in Table 3, treatment of GO and DADS resulted in a significantly (*P* < 0.001) decreased of serum MDA, GSHPx, GSH, and GSSG levels in comparison to the control group. Further, HFD resulted in significant (*P* < 0.001) increase in MDA, GSHPx, GSH, and GSSG levels compared to NFD. In addition, compared to a HFD control, five days of GO and DADS treatment reduces elevated levels of MDA, GSHPx, and GSH in serum. Furthermore, both GO, and a high dose of DADS significantly (*P* < 0.001) reduced serum GSSG levels in comparison to HFD control.

**Table 3:** Oxidative parameters.

Groups	MDA	GSHPx	GSH	GSSG
NC	0.47 ± 0.01	0.52 ± 0.01	5.89 ± 0.23	4.61 × 10^-2^ ± 0.1 × 10^-2^
NGOL	0.39 ± 0.02*	0.45 ± 0.03*	5.23 ± 0.38*	4.03 × 10^-2^ ± 0.2 × 10^-2*^
NGOH	0.31 ± 0.03^**^	0.32 ± 0.03^**^	3.99 ± 0.32^***^	3.21 × 10^-2^ ± 0.2 × 10^-2**^
NDADSL	0.43 ± 0.03	0.47 ± 0.04*	4.98 ± 0.248^**^	4.11 × 10^-2^ ± 0.3 × 10^-2**^
NDADSH	0.35 ± 0.02*	0.35 ± 0.06^**^	4.76 ± 0.31^**^	3.38 × 10^-2^ ± 0.4 × 10^-2***^
HFDC	0.86 ± 0.06^aaa^	0.78 ± 0.02^aaa^	7.81 ± 0.36^aaa^	7.92 × 10^-2^ ± 0.1 × 10^-2aaa^
HFGOL	0.72 ± 0.07*	0.68 ± 0.07*	6.59 ± 0.39*	6.11 × 10^-2^ ± 0.1 × 10^-2*^
HFGOH	0.51 ± 0.08^**^	0.41 ± 0.05^***^	5.60 ± 0.44^**^	4.86 × 10^-2^ ± 0.2 × 10^-2**^
HFDADSL	0.68 ± 0.10*	0.69 ± 0.03*	5.84 ± 0.34^**^	6.29 × 10^-2^ ± 0.1 × 10^-2*^
HFDADSH	0.56 ± 0.09^**^	0.43 ± 0.02^**^	4.78 ± 0.26^***^	4.91 × 10^-2^ ± 0.5 × 10^-2***^

Values are given as mean ± SEM of eight rats; diallyl disulfide (DADS); NC: normal control; NGOL: normal Garlic oil low dose (50 mg/kg); NGOH: normal Garlic oil high dose (100 mg/kg); NDADSL: normal DADS low dose (4.47 mg/kg); NDADSH: normal DADS high dose (8.94 mg/kg); HFDC: high fat diet control; HFGOL: high fat Garlic oil low dose (50 mg/kg); HFGOH: high fat Garlic oil high dose (100 mg/kg); HFDADSL: high fat DADS low dose (4.47 mg/kg); HFDADSH: high fat DADS high dose (8.94 mg/kg); Melondialdehyde: MDA (nmol/ml); glutathione peroxidase: GSHPx (U/mg protein); reduced glutathione: GSH (nmol/ml); oxidized glutathione: GSSG (nmol/ml); *P < 0.05; ^**^P < 0.01 and ^***^P < 0.001 normal diet fed treated groups Vs normal fat diet control and high fat diet treated Vs high fat diet control respectively. ^aaa^*P* < 0.001 NFD control Vs HFD control.

### 3.4 Impact on antioxidant profile

As evident from Table 4, high doses of GO and DADS increased SOD and CAT activities in LTH in comparison to NFD control. Additionally, FRAP and total SH values were significantly (*P* < 0.001) elevated in LTH of rats exposed to GO (both doses) and a high dose of DADS. In comparison to a normal control, both GO, and DADS (high dose) reduced TBARS levels in LTH. When rats were given GO and DADS for five days in a row, the levels of antioxidants in LTH increased from deficient to normal. Furthermore, when animals were given GO and DADS, their TBARS levels returned to normal.

**Table 4:** Antioxidant parameters.

Groups	SOD	CAT	TBARS	FRAP	SH group
NC	82.4 ± 1.5	68.3 ± 1.7	34.89 ± 1.2	4.38 ± 0.4	0.58 ± 0.02
NGOL	87.7 ± 1.3	78.6 ± 1.4*	27.10 ± 1.3*	4.99 ± 0.2*	0.77 ± 0.06*
NGOH	96.6 ± 1.5*	99.2 ± 1.5^**^	21.21 ± 2.2^***^	6.29 ± 0.3^***^	0.83 ± 0.01^**^
NDADSL	86.5 ± 1.7	83.7 ± 1.4*	30.33 ± 2.1	5.05 ± 0.2^**^	0.74 ± 0.02*
NDADSH	93.4 ± 1.5*	95.2 ± 1.4^**^	24.27 ± 1.8^**^	5.98 ± 0.4^***^	0.79 ± 0.01^**^
HFDC	38.1 ± 1.4^aaa^	38.3 ± 1.5^aaa^	51.22 ± 1.7^aaa^	3.23 ± 0.9^aa^	0.34 ± 0.04^aaa^
HFGOL	71.3 ± 1.5^***^	59.5 ± 1.6^**^	41.29 ± 1.8*	3.66 ± 0.5	0.54 ± 0.03*
HFGOH	82.9 ± 1.4^***^	78.3 ± 1.8^***^	28.19 ± 1.2^**^	5.03 ± 0.2^**^	0.66 ± 0.03^***^
HFDADSL	68.3 ± 1.8^**^	65.2 ± 2.1^**^	43.76 ± 1.1*	4.23 ± 0.4*	0.59 ± 0.02*
HFDADSH	78.4 ± 1.2^***^	75.1 ± 2.2^***^	31.54 ± 1.8^**^	4.85 ± 0.1^**^	0.69 ± 0.02^***^

Values are given as mean ± SEM of eight rats; diallyl disulfide (DADS); NC: normal control; NGOL: normal Garlic oil low dose (50 mg/kg); NGOH: normal Garlic oil high dose (100 mg/kg); NDADSL: normal DADS low dose (4.47 mg/kg); NDADSH: normal DADS high dose (8.94 mg/kg); HFDC: high fat diet control; HFGOL: high fat Garlic oil low dose (50 mg/kg); HFGOH: high fat Garlic oil high dose (100 mg/kg); HFDADSL: high fat DADS low dose (4.47 mg/kg); HFDADSH: high fat DADS high dose (8.94 mg/kg); Superoxide dismutase: SOD (unit/g tissue); Catalase: CAT ((unit/g tissue); thiobarbituric acid reactive species: TBARS (nmol/g tissue); ferric reducing/antioxidant power: FRAP (µmol/g tissue); Total sulfhydryl: SH group (µmol/g tissue); *P < 0.05; ^**^P < 0.01 and ^***^P < 0.001 normal diet fed treated groups Vs normal fat diet control and high fat diet treated Vs high fat diet control respectively. ^aaa^*P* < 0.001 NFD control Vs HFD control.

## 4 Discussion

This study was done to evaluate the beneficial role of GO and its active constituent, DADS, in obviating dyslipidemia using experimental animals. The findings suggest that both GO, and DADS have the capacity to reduce hyperlipidemia-induced oxidative stress, with GO having a slight advantage. These findings suggest that additional GO elements may be contributing therapeutic value to the DADS found in GO, and that DADS alone is not attributable for GO's pharmacological effect in preventing dyslipidemia.

There was an increase in TG, LDL, and TC levels in high fat diet animals. The serum TG levels were reduced after treatment with GO and DADS. This trend could be attributable to increased release of endothelium-bound lipoprotein lipase, that causes hydrolysis of TG into fatty acids, mediated by GO and DADS (Devi and Sharma, 2004). A high amount of TC, specifically LDL cholesterol, is triggering factor for coronary artery disease. LDL is responsible for cholesterol buildup in the arteries and aorta, which leads to coronary heart disease (De Graat et al., 2002). Both high and low doses of GO, as well as a high dose of DADS, significantly reduce TC and LDL, implying that GO and DADS have cardioprotective properties. The increased level of cardioprotective lipoprotein HDL in the HFD group after giving high doses of GO and DADS could be responsible for an increase in the activity of the LCAT, which helps regulate blood lipids. Several studies have linked an increase in HDL to a decreased risk of coronary artery disease (Wilson, 1990), and most medications that reduces TC also lower HDL. In the current study, however, both GO, and DADS reduced TC and LDL while significantly increasing HDL, demonstrating their superiority to alternative hypolipidemic.

Hyperlipidemia causes hepatocyte membrane damage, allowing endogenous enzymes to enter the bloodstream (Kew, 2000, Bolkent et al., 2004). As a result, liver enzymes leaked into the serum, however, GO and DADS decreases the level of this enzymes in the blood indicating their protective effect. Both GO and DADS significantly restored normal enzyme levels in the serum, implying that hepatocytes are protected by efficiently decreasing lipid levels.

The production of reactive free radicals is well known because of hyperlipidemia-induced stress. Despite normal mRNA expression, a minor increase in hydrogen peroxide causes inactivation of CAT and GSH (Aronoff, 1965, Beazley et al., 1999). Hydrogen peroxide breakdown is catalyzed by catalase and glutathione peroxidase enzymes. We know that H_2_O_2_ concentrations are more at the site the damage, and that H_2_O_2_ penetration into plasma is less. The neutralization of hydrogen peroxide in blood is simplified by GSHPx activities. The breakdown of erythrocytes is one source of plasma antioxidant enzymes. Elevated GSHPx activity could be a result of erythrocyte lysis caused by reduced GSHPx activity and high reactive oxygen species levels in the RBC. Oxidative stress is caused by an increase in free radicals due to H_2_O_2_ production and an imbalance in the oxidant/antioxidant balance, which causes oxidative damage and an elevation in MDA levels (Cohen and Hochstein, 1964). In the current investigation, we discovered that GO and DADS both depleted the rise of MDA, GSHPx, GSH, and GSSG in serum, exhibiting significant free radical scavenging effect.

In pathological situations including hyperlipidemia, there is imbalance between oxidant and antioxidant systems (Parihar and Hemnani, 2003, Abdollahi et al., 2004). As a result, we used the FRAP assay, that revealed a remarkably diminished free radical scavenging property of GO and DADS, in hyperlipidemic rats. SH groups are involved in a variety of biochemical and metabolic processes in the body, in addition to activation of antioxidant enzymes (Jansen, 1959). Also, it is established that t is vulnerable to free radical damage and gets lowered at the time of stress such as dyslipidemia. In liver tissues, GO and DADS had higher SH levels than the HFD control, indicating that they aid in recovering the pool of SH group.

As previously stated, GO and DADS are both excellent antioxidants and anti-hyperlipidemic agents. However, the protective action of GO (high dose) was more than DADS (high dose). DADS has therapeutic benefits as a principal/major constituent of GO; however, the outcome of this study demonstrate that the DADS is not the only element of GO for all its biological benefits. This is supported by the fact that a high dose of GO has a greater therapeutic benefit than a high dose of DADS.

## 5 Conclusion

Garlic and its main active constituent, diallyl disulphide were efficient in preventing dyslipidemia and neutralizing reactive free radicals caused by a high fat diet in hyperlipidemic rats. The fact that GO has a greater therapeutic impact than its active ingredient, DADS, is an interesting finding. These observations show that, in addition to GO's DADS, other elements are responsible for the compound's synergistic antihyperlipidemic and antioxidant properties.

## Funding

Syed Mohammed Basheeruddin Asdaq would like to express his gratitude to AlMaarefa University, Riyadh, Saudi Arabia, for providing funding (MA-004) to carry out this study.

## Declaration of Competing Interest

The authors declare that they have no known competing financial interests or personal relationships that could have appeared to influence the work reported in this paper.
